# Setting the stage for Cancer Advocacy in Africa: how?

**DOI:** 10.1186/1750-9378-8-S1-S6

**Published:** 2013-07-15

**Authors:** Folakemi T Odedina, Belmira Rodrigues, Priya Raja

**Affiliations:** 1 Pharmaceutical Outcomes and Policy, College of Pharmacy, University of Florida, Gainesville, FL, USA; 2 African Organisation for Research and Training in Cancer (AORTIC), Cape Town, South Africa; 3 University of Chicago Medical Center, Chicago, IL, USA

## Abstract

**Background:**

Oftentimes, cancer advocates in Africa look at the developed nations in North America and Europe for guidance on cancer advocacy. However, lessons learnt from developed nations do not necessarily apply to the situational context of Africa. Without a doubt, successful cancer advocates in Africa can best serve as learning sources and role models for advocacy in Africa. This paper describes the results of an environmental scan of advocacy organizations in Africa.

**Methods:**

A cross-sectional study design was employed for this project. Using a structured survey data collection form, participants submitted their responses either by online submission (Google docs) or by electronic mail to admin@aortic-africa.org.

**Results:**

A total of 39 African advocates representing 17 countries participated in the project. The majority of participants have been advocates for more than five years; and mostly advocate for both males and females and individuals between the ages of 30 and 39. The most common cancers focused on by the advocacy organizations include breast, prostate, liver, cervix, stomach, bladder, pediatric, colorectal and neck. The information provided by participants offer clear guidelines on establishing and maintaining an advocacy program in Africa despite the various challenges faced by these organizations.

**Conclusion:**

Whilst this paper only highlights a subset of advocacy initiatives on the Continent, there is an opportunity for a more inclusive dialogue for advocates to share ideas with each other, connect with other advocates, learn about other innovative advocacy programs, and join the global war against cancer. To this end, the **biennial *International Workshop on Cancer Advocacy for African Countries* (*CAAC*)** during the next **AORTIC International Cancer conference,** offers an opportunity to further Africa’s cancer advocacy initiatives.

## Introduction

"In Africa, literally every day, hundreds, possibly thousands of people die needlessly in pain from cancer for want of pain relief that could cost literally pennies rather than pounds. The basic infrastructure and resources to cope with the new health epidemic is basically not there and we have to do something about it. We know that there is a steam train that is coming down the track and we have a choice - we can wither, build some new track or we can wait for the train to hit us."

Former British health minister Alan Milburn’s statements while chairing the conference on the geography of cancer in Africa (2007).

The need for cancer advocacy in Africa is indisputable. Advocacy is widely seen as the *new track* needed to stop the *stream train* of cancer. Defined as the “*effort to influence people*, *primarily decision-makers*, *to create change*, *which in the context of cancer control results in comprehensive policies and effective program implementation*, *through various forms of persuasive communication*”,[1] cancer advocacy is in its infancy in Africa. This is rather unfortunate given that successful national cancer control planning and implementation cannot take place without advocacy. Active advocacy will be pivotal to turn the tide on the cancer crisis and make issues related to the disease a high priority in Africa.

Although cancer advocacy is not widespread in Africa, there are some individuals who are deeply committed to fighting the disease in Africa, even with limited resources and support. These individuals work tirelessly to conquer cancer within their communities, countries and the continent by mobilizing the limited resources within Africa for cancer control, partnering with key stakeholders to increase awareness about the disease, fundraising to support their activities, and implementing programs sensitive to the needs of their communities. Cancer advocates in Africa are the ***everyday heroes*** of Africa.

In general, new cancer advocates in Africa look at the developed nations in North America and Europe for guidance on cancer advocacy. However, lessons learnt from developed nations often are not applicable to the circumstances in developing countries due to the significant differences in the situational context. Given that some (although few) cancer advocates have successfully implemented advocacy programs in Africa, sometimes in spite of and under adverse conditions, these advocates can best serve as learning sources and role models for advocacy in Africa. The African Organisation for Research and Training in Cancer (AORTIC) thus conducted an environmental scan of the African continent to showcase these outstanding cancer advocacy practices by various organizations in Africa.

## African Organisation for Research and Training in Cancer (AORTIC)

AORTIC is Africa’s pre-eminent non-profit organization working for cancer control. AORTIC achieves its mission not only by facilitating research and training, but also by providing relevant and accurate information on the prevention, early diagnosis, treatment, and palliation of cancer. A key interest of the organization is advocacy, as it is dedicated to increasing public awareness of cancer and reducing the stigma associated with it. With membership from countries throughout Africa and the international cancer community, the main objectives of AORTIC are to further research related to cancers prevalent in Africa, enable and support training initiatives in oncology for health care workers, create cancer prevention and control programs, and to raise public awareness of cancer on the continent. AORTIC strives to unite the African continent in achieving its goal of a cancer-free Africa and seeks to make a positive impact throughout the region through collaboration with health ministries and global cancer organizations.

One way in which AORTIC achieves its objectives is by promoting advocacy in Africa. Thereby, in line with the mission of AORTIC, the primary objectives of this study were to: (i) describe the types of cancer advocacy being practiced in Africa and (ii) identify essential recommendations for new cancer advocates and those considering being involved in cancer advocacy.

## Methods

We employed a cross-sectional study design using a structured survey data collection form. The data collection form (see Additional file 1) was developed by investigators and comprised both open- and close-ended questions. The form started with a request for background information on the cancer advocacy organization and the advocate, including how long the respondent has been an advocate, the average age of the clients, the primary gender targeted by the organization, and the types of cancer targeted. Next, participants were asked to describe their cancer advocacy activities, including the types of advocacy practiced by the organization - political, support, fundraising, community outreach, education, and/or research. They also listed the top three recommendations they would give to someone trying to establish an advocacy program.

The face validity of the data collection form was established by two African-American cancer advocates in the United States. We did not ask cancer advocates in Africa to pretest the survey because we did not want to lose potential participants for the project. The two advocates who pre-tested the data collection form were asked to read and complete the survey. Subsequently, they were contacted and asked to provide feedback on the form, including comprehension, ease of administration, and the length of completion of the survey. Following the pretest, no changes were made as the two advocates expressed that the data collection form was appropriate for the environmental scan project. It is worth noting that employing advocates in the United States for the face validation of the data collection form is a potential limitation given the differences between North American and African countries. However, participants of this project did not indicate any difficulty in completing the data collection form. While we are cognizant of the fact that Africa is multi-lingual, the data collection form was in English only. In subsequent studies, consideration will be given to administer the survey in English and French.

A convenient but targeted sampling method was employed to recruit participants as follows: (i) electronic mails sent to members of AORTIC; (ii) electronic mails sent to 51 members of the African Cancer Advocates Consortium; and (iii) survey information provided on the AORTIC website. Participants submitted their responses either by online submission (Google docs) or by electronic mail to admin@aortic-africa.org. A database of all responses was compiled in an excel spreadsheet and descriptive analysis was used to summarize the characteristics of the advocacy organizations and the types of cancer advocacy in Africa. From the open-ended responses, we summarized the essential recommendations for cancer advocacy in Africa.

## Results

The first wave of data collection ended on Friday, August 17, 2012. There were a total of 42 responses with three responses from advocates outside Africa. The three non-African responses were excluded from the data analyses. The 39 African advocates represented 17 countries, with the majority of the participants from Nigeria (28.21%). The 17 countries represented in this survey are: Nigeria (11 participants); Uganda (3 participants); Kenya (3 participants); Egypt (3 participants); South Africa (3 participants); Guinea (2 participants); Tanzania (2 participants); Ghana (2 participants); Sierra Leone (2 participants); Ethiopia (1 participant); Sudan (1 participant); Algeria (1 participant); Mali (1 participant); Namibia (1 participant); Malawi (1 participant); Zambia (1 participant); and Zimbabwe (1 participant). Table 1 provides a summary of the characteristics of the participants. The majority of participants have been advocates for more than five years, and both males and females between the ages of 30 and 39 are targeted by their organizations. It is interesting to note that most of the advocacy organizations focus on Education Advocacy while only a few focus on Political Advocacy. The participants target a range of cancer types. However, the most common cancers are breast, prostate, liver, cervix, stomach, bladder, pediatric, colorectal and neck. About 25% of the participants indicated that their organizations focus on all types of cancer, while about 10% indicated focus on tobacco-related cancers.

**Table 1:** Characteristics of Participants (N=39)

Question	Responses	Frequency	Percentage
How long have you been an advocate?	Within past year (anytime less than 12 months ago)	**4**	**10.26%**
	
	Within past 2 years (1 year but less than 2 years ago)	**1**	**2.56%**
	
	Within past 5 years (2 years but less than 5 years ago)	**9**	**23.08%**
	
	5 or more years ago	**25**	**64.10%**

			

What is the age of the population you serve? (Choose all that applies)	Between 30 and 39 years	**27**	**69.23%**
	
	Between 40 and 49 years	**25**	**64.10%**
	
	Between 50 and 59 years	**19**	**48.72%**
	
	Between 60 and 69 years	**13**	**33.33%**
	
	Between 70 and 79 years	**8**	**20.51%**
	
	80 years or above	**5**	**12.82%**

			

What is the primary gender that you target?	Male only	**0**	**0%**
	
	Female only	**4**	**10.26%**
	
	Both male and female	**35**	**89.74%**

			

What types of advocacy does your program or organization focus on? (Choose all that applies)	Political	**17**	**43.59%**
	
	Support	**25**	**64.10%**
	
	Fundraising	**18**	**46.15%**
	
	Community Outreach	**27**	**69.23%**
	
	Education	**36**	**89.74%**
	
	Research	**28**	**71.79%**
	
	Other	**0**	**0%**

### Identification of community needs

One of the primary factors critical to successful advocacy is to meet the needs of the community. Participants were asked how they identify community needs for their advocacy programs. A participant from Algeria reported the identification of community needs through the statistical trend of cancer incidence, survival, and stage of cancer. In Uganda, an advocacy organization employs needs assessment surveys and insights from community outreach events to identify the needs of the community. It was encouraging to see that almost half of the participants reported conducting needs assessment for their targeted communities. Other methods employed by participants to identify community needs are:

• Hospital records.

• Ministry of Health records.

• Scientific journal publications.

• Technical reports.

• Clinician observations.

• Research data from local institutions.

• Patient feedback from health events and by direct contact with the organization.

• Interactions during community outreach.

• Support group meetings.

• Feedback from Community Health Workers.

### Mission and key goals of advocacy programs

The advocacy mission and goals of participants’ organizations ranged from broad initiatives to well-focused agendas, reflecting the needs of the target population. For example, the goals of an advocacy organization in Tanzania reflect a mission to address tobacco-related cancers and include:

• To facilitate the co-ordination of tobacco control activities in Tanzania.

• To undertake tobacco control activities and campaigns in Tanzania.

• To persuade the Tanzanian government to ratify and fully implement the WHO Framework Convention on Tobacco Control (WHO FCTC) Treaty.

• To improve implementation of the Tobacco Products (Regulations) Act 2003 and such other laws that seeks to control and stop the use of tobacco in Tanzania.

• To raise public awareness on the hazards of tobacco use.

• To document and publicize information on tobacco control in Tanzania.

• To promote research on alternative crops in tobacco growing areas and, assist in securing markets for such crops.

• To maintain close working relationships with government institutions, parliamentarians, the private sector and all tobacco control stakeholders, with the aim of mounting effective advocacy campaigns against tobacco use in Tanzania.

The outcomes provided by this organization were: (i) a strong and well organized members' forum and network; (ii) a society (particularly adults) aware of the effects of tobacco use; (iii) a supportive society; and (iv) farmers adopting alternative crops to tobacco (more than 70% in southern Tanzania).

On the other hand an advocacy organization in Ethiopia focused on addressing cancer issues through public health initiatives with the following goals:

1. Encourage policy dialogue among decision makers for the formulation of legislations and implementation of different public health issues

2. Actively work on the ratification and implementation of the WHO-Framework Convention on Tobacco Control (FCTC).

3. Actively contribute towards the development of directives to control tobacco products in Ethiopia by the Food Medicine and Health Care Administration and control Authority.

4. Undertake research gap assessment and prioritize research topics.

5. Conduct research and public health evaluations based on priority health issues.

6. Identify areas for review and promotion of policy development.

Instead of targeting a specific cancer, an advocacy organization in Nigeria focuses on political advocacy and aims to: (i) Sensitize all levels of government on the importance of establishing excellent cancer services and networks; and (ii) Partner with governments, professionals and other non-governmental organizations (NGOs) in health education related to cancer.

The expected outcomes identified by this Nigerian organization are a society well informed about cancer, government provision of good preventive and curative cancer services, and networks supported by professional associations, NGOs and other society groups.

In Egypt, an advocacy organization focused on women empowerment with the goals of:

1. Eliminating cultural taboos of breast cancer and breast health.

2. Breaking the wall of fear and silence between doctors and the community.

3. Empowering women with the knowledge to take action and help them make informed decisions about treatment.

4. Developing media campaigns to encourage men to get their women screened and to support them if they are diagnosed with breast cancer.

In South Africa, one of the leading cancer advocacy organizations was formed to address health equity for cancer patients in the public sector, with specific goals to:

1. Improve access to treatment for all cancer patients.

2. Ensure psycho-social support becomes part of patient-centered care in oncology.

3. Promote early detection and cancer awareness.

4. Promote the right to a second opinion in oncology for patients.

5. Ensure equity of cancer treatment.

6. Advocate for re-employment of patients.

7. Address stigma issues about cancer.

8. Advocate for a population-based cancer registry and National Cancer Plan.

A Ugandan advocacy organization innovatively assesses the outcomes of the program aims to track their progress. The aims of the advocacy program are to: (i) Advocate for the development and implementation of policies favoring control and management of cancer; (ii) Establish the prevalence of breast and cervical cancer in Uganda; (iii) Lobby government and other stakeholders to increase financial and other resources by 50%; and (iv) Intensify breast and cervical cancer awareness campaigns on early detection, diagnosis treatment and provide support services.

At the time of this survey, the outcomes documented for the Ugandan advocacy organization include: (i) increase in the number of women taking action for early detection; (ii) involvement of the government in cancer prevention in the country; (iii) membership affiliation with the Union for International Cancer Control (UICC) and International Alliance for Patient Organisation (IAPO); (iv) formation of two umbrella organizations, the Uganda Cancer Society and the Uganda Non- Communicable Diseases Alliance; and (v) improvement in cancer services in the country.

### Recommendations for establishing and sustaining an advocacy program

Finally, participants were asked to list the top recommendations for establishing an advocacy program in Africa. Some of the recommendations provided by participants included:

• Conduct situational analysis to define the community and conduct community needs assessment.

• Develop a strategic plan that includes, vision, mission, type of advocacy that will be implemented, feasible and achievable goals, and strategies for implementing goals.

• Set an action plan of evaluation and impact assessment that will guarantee the sustainability of the advocacy plan.

• Select the advocacy issue of concern to the community and design an appropriate strategy to effectively address the concern.

• Invest in a network of dedicated and committed volunteers as part of the workforce.

• Identify and establish good relationship with all key stakeholders, including the policy makers, other NGOs, political leaders and health authorities.

• Work closely with the media in promoting programs.

• Get close to the people the organization wants to advocate for, understand what they are going through and let them work with the organization in helping to lead the way.

• Partner with experienced professionals and build a network of good will.

• Employ scientific data to develop and support program activities.

• Knowledge is power, so it is essential to continuously learn about cancer and advocacy issues that will affect the program.

• Maintain a positive attitude towards the community and the issue.

• Ensure that everyone representing the organization is experienced and capable of dealing with the advocacy issues.

• Be aware of advocacy resources and tools by organizations such as the World Health Organization (WHO) and AORTIC.

## Conclusion

This paper is the first effort of an environmental scan of cancer advocacy organizations to understand the current status of cancer advocacy in Africa. Although cancer advocacy in Africa may not be at the same level as seen in Europe and North America, there are dedicated African advocates who passionately employ innovative ways to address cancer in Africa. Two advocacy areas that need attention are Political Advocacy and Fundraising Advocacy. Less than 20% of the organizations who participated in this environmental scan focus on these advocacy types. While a concerted effort must be made to train advocates in all areas of advocacy, special attention needs to be paid to the neglected areas of Political and Fundraising Advocacy.

The information provided by participants offer clear guidelines to establishing and sustaining an advocacy program in Africa, despite the challenges faced by advocates. Whilst this paper only highlights a subset of advocacy initiatives on the Continent, there is an opportunity for a more inclusive dialogue for advocates to share ideas with each other, connect with other advocates, learn about other innovative advocacy programs, and join the global war against cancer. To this end, the **biennial *International Workshop on Cancer Advocacy for African Countries* (*CAAC*)** during the next **AORTIC International Cancer conference,** offers an opportunity to further Africa’s cancer advocacy initiatives. The 2013 workshop will include an ***Advocacy Expo & Posters*** session, offering participants the chance to share, connect, network, discover and learn from each other. Interested participants can join fellow cancer advocates, survivors, scientists, clinicians, and policy makers during the AORTIC 2013 International Cancer Conference entitled “Cancer in Africa: Bridging Science & Humanity” in Durban, South Africa (http://www.aortic2013.org).

## List of abbreviations used

AORTIC: African Organisation for Research and Training in Cancer; CAAC: Cancer Advocacy for African Countries; NGOs: Non-Governmental Organizations; WHO: World Health Organization; WHO FCTC: WHO Framework Convention on Tobacco Control.

## Competing interests

The authors declare they have no competing interests.

## Authors’ contributions

FTO developed the project, developed the items for the data collection instrument, conducted the analyses and wrote the manuscript. BR coordinated the data collection and contributed to the manuscript. PR assisted with the development of the initial draft of data collection form, designed the data collection form and collected data and conducted the data entry.

## Supplementary Material

Additional file 1 Data collection instrument is attached as Appendix I. Click here for file

## Declarations

The authors declare that funding for publication of the article was funded by an award provided by the National Cancer Institute (Award Number R13CA162899).

This article has been published as part of *Infectious Agents and Cancer* Volume 8 Supplement 1, 2013: Selected articles from The International Workshop on Cancer Advocacy for African Countries (CAAC) 2011. The full contents of the supplement are available online at http://www.infectagentscancer.com/supplements/8/S1 The Supplement Editors declare they have no competing interests.
