# Supplementary material for: Setting the stage for Cancer Advocacy in Africa: how?
Source: Infect Agent Cancer. 2013 Jul 15;8(Suppl 1):S6. doi: 10.1186/1750-9378-8-S1-S6 (PMC3716680; doi:10.1186/1750-9378-8-S1-S6)
Supplement: Additional file 1 — Data collection instrument is attached as Appendix I. [file 1750-9378-8-S1-S6-S1.pdf]

Appendix I
Africa Cancer Advocacy Toolkit Supplement Case Study Collection Form

1. Background Information

Organization Name	

Organization Address (Mailing address, city, country, and postal code)	

Your Name	

Your Title / Role in the Organization	

Your Phone #	

Your Email address	

How long have you been an advocate?	☐ Within past year (anytime less than 12 months ago)<br>☐ Within past 2 years (1 year but less than 2 years ago)<br>☐ Within past 5 years (2 years but less than 5 years ago)<br>☐ 5 or more years ago

What is the average age of the population you serve?	☐ Between 30 and 39 years ☐ Between 40 and 49 years<br>☐ Between 50 and 59 years ☐ Between 60 and 69 years<br>☐ Between 70 and 79 years ☐ 80 years or above

What is the primary gender that you target?	☐ Male only ☐ Female only<br>☐ Both male and female

What types of cancer do you target? (List all)	

2. Cancer Advocacy Information & Activities

What types of advocacy does your program or organization focus on? (Choose all that applies)

- [ ] Political
- [ ] Support
- [ ] Fundraising
- [ ] Community Outreach
- [ ] Education
- [ ] Research
- [ ] Other: Please state ______________________

Why was your organization formed?

How did you begin setting up your advocacy network?

What issues does your organization focus on in general? What are the mission and key goals?

What are the key aims and outcomes for your advocacy program? How did you develop these?

How did you identify the needs of the community?

Please list the top three recommendations you would give to those trying to establish an advocacy program.

3. Cancer Advocacy Case Study

Describe a political, education, fundraising, community outreach, research, or support advocacy that **you successfully worked on within the last year**, providing comprehensive details on key aims, methods, actors, timeframe, and outcomes. In addition, provide a **Photovoice narrative** about this advocacy activity. A Photovoice strategy is a way for people to represent their community activities. For the Photovoice narrative, provide at least one photograph (maximum of three photographs) and describe the elements of the photograph(s) and their meaning in relationship to cancer advocacy.

Please describe the key ingredients that have made your organization and its advocacy work to be successful.

What were some key problems or obstacles that you faced in your advocacy work? Please provide at least one example of how you overcame an obstacle in advocacy.

If you have any additional commentary or information related to advocacy work that you would like to provide, please use the space below to do so.
